# Multivariate analysis of ultrasound-recorded dorsal strain sequences: Investigation of dynamic neck extensions in women with chronic whiplash associated disorders

**DOI:** 10.1038/srep30415

**Published:** 2016-08-03

**Authors:** Anneli Peolsson, Gunnel Peterson, Johan Trygg, David Nilsson

**Affiliations:** 1 Department of Medical and Health Sciences, Division of Physiotherapy, Linköping University, Sweden; 2 Centre for Clinical Research Sörmland, Uppsala University, Sweden; 3 Computational Life Science Cluster (CLiC), Department of Chemistry, Umeå University, Sweden

## Abstract

Whiplash Associated Disorders (WAD) refers to the multifaceted and chronic burden that is common after a whiplash injury. Tools to assist in the diagnosis of WAD and an increased understanding of neck muscle behaviour are needed. We examined the multilayer dorsal neck muscle behaviour in nine women with chronic WAD versus healthy controls during the entire sequence of a dynamic low-loaded neck extension exercise, which was recorded using real-time ultrasound movies with high frame rates. Principal component analysis and orthogonal partial least squares were used to analyse mechanical muscle strain (deformation in elongation and shortening). The WAD group showed more shortening during the neck extension phase in the trapezius muscle and during both the neck extension and the return to neutral phase in the multifidus muscle. For the first time, a novel non-invasive method is presented that is capable of detecting altered dorsal muscle strain in women with WAD during an entire exercise sequence. This method may be a breakthrough for the future diagnosis and treatment of WAD.

One year after whiplash injury, up to half of affected individuals have remaining neck pain due to whiplash associated disorders (WAD)^1^. Morphological muscle changes^2^,^3^, altered muscle behaviour^4^,^5^,^6^, and decreased endurance of the neck muscles^7^,^8^ are features of WAD. Degenerated and deconditioned neck muscles may affect the physical support of the cervical spine^9^ and are significant factors for remaining pain and disability^10^; although, they can be improved with neck-specific exercises^8^,^11^,^12^. As there is no consensus on the injury mechanism or the best treatment in chronic WAD, more knowledge of muscle behaviour is needed for future improved care and to determine the best therapeutic exercises. The behaviour of the ventral neck muscles in neck pain patients has frequently been investigated^5^,^6^,^13^,^14^,^15^. However, little is known about the role of the dorsal neck muscle layers during dynamic loaded neck exercises^16^. Peolsson *et al*.^16^ investigated multilayer dorsal neck muscles with ultrasonography during the range of standardised dynamic low-loaded neck extension in an upright seated position, where postural control is vital. The study by Peolsson *et al*.^16^ showed altered activation in the semispinalis capitis in patients with chronic cervical disc disease compared with healthy controls. The behaviour of the different dorsal neck muscle layers during dynamic neck extension has not been investigated in individuals with chronic WAD and no one has investigated multilayer muscle behaviour registered with ultrasound movies during the entire sequence of a neck movement. The aim of this study was to compare the strain (the degree of muscle deformation expressed as the percentage of longitudinal shortening or elongation during the entire exercise compared with the start of the exercise/“rest”) of dorsal multilayer neck muscles in individuals with chronic WAD and matched healthy controls, during a standardised dynamic resisted neck extension. Non-invasive ultrasonography movies, post-process speckle tracking, and advanced multivariate statistics were used to investigate the real time mechanical muscle behaviour during the entire sequence of neck extension.

## Methods

### Design

This is an experimental cross-sectional age and gender matched controlled study comparing women with chronic WAD and healthy controls.

### Participants

This study included nine women (aged 38 ± 11.3 [mean ± SD] years) with chronic WAD (mean time since injury 21 ± 5.1 months). Subjects had a neck-specific disability rated as 15 ± 4.5 on the Neck Disability Index (NDI) (0 = no disability, 50 = highest score for disability), an average pain intensity during the last week of 46 mm on a Visual Analogue Scale (VAS) (0 = no pain, 100 = worst imaginable pain), and a Body Mass Index (BMI) of 24 ± 4.2. Age and gender matched controls had a mean age of 38 ± 11.6 years, NDI of 0.8 ± 1.1, pain intensity of 1  ± 1.3 mm on a VAS, and a BMI of 23.6 ±  2.7.

All subjects and controls voluntarily participated in the study. Participants were consecutively recruited and volunteered for ultrasound investigation at baseline from a larger multi centre randomised controlled study^17^. Eligibility criteria for the randomised controlled study were: chronic WAD grade 2 (patients’ neck symptoms verified to originate from the neck and from a whiplash injury) and 3 (same as WAD grade 2, but with additional neurological findings of cervical radiculopathy)^18^, aged 18–63 years, a whiplash injury in the preceding 6–36 months that was nominated as the cause of current symptoms, a NDI^19^ of at least 10/50, and/or an average pain on a VAS of >20/100 mm (0 = no pain, 100 = worst imaginable pain)^20^ for the preceding week. Exclusion criteria were signs of traumatic brain injury, earlier fracture or luxation of the cervical spine, neck pain causing a work absence longer than one month in the year preceding the whiplash injury, myelopathy, spinal infection/tumour/previous neck surgery, more dominant pain elsewhere in the body, conditions potentially detrimental to completing the study interventions, or insufficient knowledge of the Swedish language. Additional criteria for the ultrasound study were right-handedness with dominant right-sided symptoms and living close to the hospital where the neck device (DBC 140, not portable) was located. All subjects who were asked to participate volunteered to be included.

The healthy controls were recruited from hospital staff, university staff, and acquaintances. Exclusion criteria were current or past neck problems, trauma to the neck or head including WAD, low back pain, neurologic or rheumatologic disease, or generalised myalgia. The regional ethics committee at Linköping University, Sweden approved the study and the Helsinki declaration was followed. The methods were carried out in accordance with the approved guidelines. All participants gave their written informed consent. No serious adverse event was reported. There were no conflicts of interest.

### Test procedure

**Figure 1:**
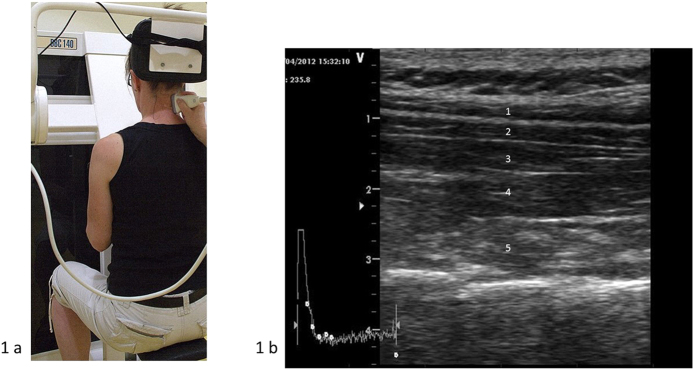
(**a**) The DBC 140 neck device. (**b**) The dorsal neck muscles: 1 = Trapezius, 2 = Splenius capitis, 3 = Semispinalis capitis, 4 = Semispinalis cervicis, 5 = Multifidus.

Subjects performed a low-loaded standardised neck extension and then returned to neutral position (0°−20°−0°) using a DBC 140 neck device (David Back Clinic International, Vantaa, Finland). Before measurements were taken, the DBC 140 seat and chest supports were adjusted for each individual and participants were carefully informed about the procedure and to cease the test if presenting neck symptoms increased. Participants were seated in the device with a straight back and neutral position of the head and neck with the seat height adjusted so that the back of the head fitted on the cushion, the chest support was tightly against the chest, feet were flat on the floor, and elbows in 90° flexion with the hands holding onto the vertical columns of the machine^21^. A trigger placed at the head cushion made it possible to synchronise the neck extension exercise with the ultrasound movie for the start and stop of the exercise (Fig. 1a).

Each participant was instructed to perform a dynamic head extension of 20° (goniometer on the machine) against a standardised low loaded resistance of 1 kg and a metronome was set at 30 beats per minute to maintain a steady pace. Instructions were given to the volunteers as follows; “listen to the beat of the metronome, bend your neck gently backwards on the first beat to reach 20° neck extension on the second beat, and be back at neutral on the third beat”. The participants practiced it twice at the speed of the metronome to familiarise themselves and to ensure accurate performance. Post-process, the real time strain of the entire head extension (neck extension back and forth from neutral position; 0°−20°−0°) was calculated from the ultrasound movies. The exercise sequence was about 4 s, with a frame rate of 235 frames/s in the ultrasound recording; thus, there was an average of 946 frames for each participant’s neck extension in the speckle tracking software.

### Ultrasound measurements

The dorsal neck muscles (from the most superficial to the deepest in the vertebral column: trapezius, splenius capitis, semispinalis capitis, semispinalis cervicis, and multifidus) (Fig. 1b) were recorded at the right side of cervical segmental level 4 (C4) with a two-dimensional, B-mode ultrasound Vivid-I scanner (GE Healthcare, Horten, Norway) and a 12 MHz linear transducer (38 mm) with high frame rates of 235 frames/s during a standardised low-loaded dynamic neck extension in a seated position. The recordings were saved in AVI format as a movie of the entire neck extension exercise sequence. The C4 vertebral level was identified and an experienced physiotherapist marked the C4 spinous process after it was located by palpation. The transducer was initially positioned in a transverse orientation at the marked C4 level to permit identification of bony landmarks and the dorsal neck muscles of the target. After identification and correct placement, the transducer was rotated 90° longitudinally along the dorsal neck muscles for an optimal image and maintained for movie recordings of the dorsal neck muscles throughout the whole neck extension sequence. The transducer placement depends on the size of the participant’s neck and muscles, approximately 2–3 cm from the spinal process/midline for optimal imaging quality of the dorsal neck muscles.The same experienced physiotherapist, with several years of ultrasound scanning, performed all the ultrasound recordings.

### Post-process speckle tracking analysis of ultrasound movies

**Figure 2:**
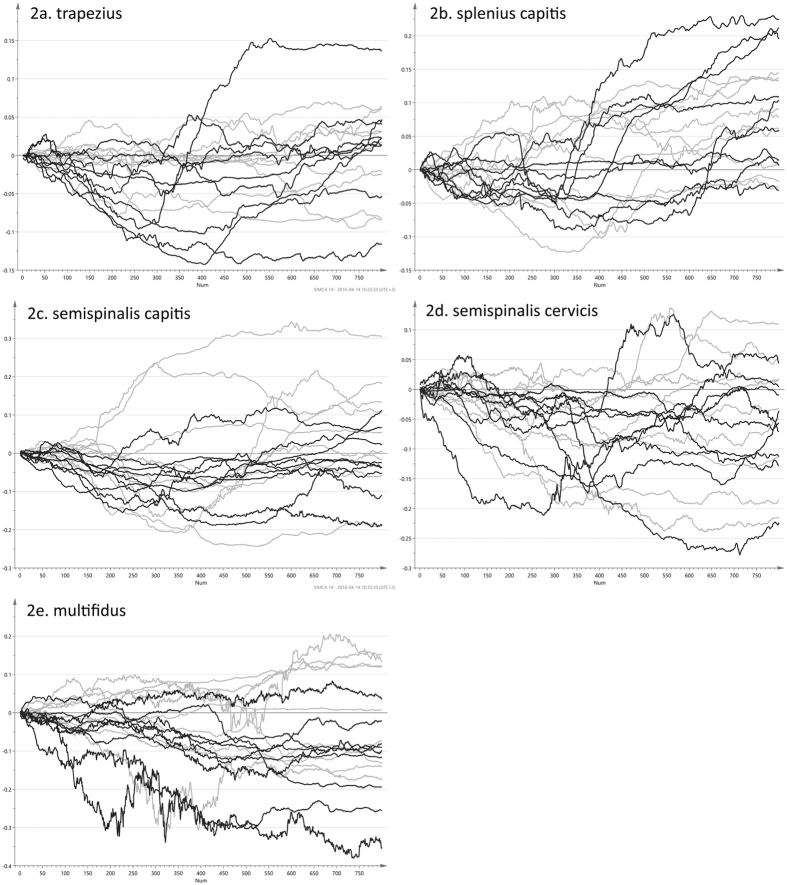
Line plots for the strain seen in the five muscle layers during the dynamic neck extension. Individuals with whiplash-associated disorders (black line) and healthy controls (light grey line). The deformation for each frame (y-axis) can be assessed by movie frame number (x-axis). (**a)** Trapezius. (**b**) Splenius capitis. (**c**) Semispinalis capitis. (**d**) Semispinalis cervicis. (**e**) Multifidus.

Real time ultrasound imaging of skeletal muscles is a reflection of sound waves with the ability to detect the unique speckle pattern (like a finger print) in the muscles. The ultrasound movies were analysed post-process by the speckle tracking method (in-house University software in Matlab) frame by frame during the entire exercise sequence, based on the Kanade-Lucas-Tomasi^22^,^23^ algorithm and further enhanced by Farron *et al*.^24^. A region of interest (ROI) is a rectangular (length of 15 mm and a width of 3.3 mm) marker manually placed in the middle of each of the five muscles of interest in the first frame of the recorded ultrasound movie. Each ROI contains a large number of measuring points and follows each muscle’s unique speckle pattern frame by frame throughout the movie of the entire exercise. When the muscles change length during exercise, so does the length of the ROI. The displacement of all measuring points within the ROI was summed and a cumulative quantitative measure of muscle strain (deformation in longitudinal shortening and elongation) was calculated in percentage (strain%) and shown as a strain sequence curve for each individual (Fig. 2a–e). The test-retest of the speckle tracking measurements used was previously reported to be reliable (Intra Class Correlation Coefficient (ICC) for the entire exercise = 0.71–0.97)^16^ and speckle tracking measurements reported to be valid^25^.

### Statistics

The multivariate methods Principal component analysis, PCA, and Orthogonal partial least squares (OPLS)^26^ were used to analyze the strain of the dorsal neck muscles during the entire exercise sequence back and forth from a seated upright neutral head position, both during the neck extension phase and return to neutral upright position.

Prior to analysis, all movies were resampled to equal length, 800 frames, which corresponds to a movie of length 3.4 s. The average length of the original movies was 946 frames with a standard deviation of 336 frames. The reason for using 800 frames for the resampling was to avoid unnecessary and excessive upscaling for some of the shorter movies. For each of the five dorsal muscles, a data set comprising the participants were assembled as five data matrices ***X***_*1*_− > ***X***_*5*_, each sized 18 × 800.

Each observation in the five matrices can be defined as a *strain sequence*, which gives the strain over the entire neck extension. Due to the extensive number of variables and also the high correlation between adjacent variables, the five matrices were subjected to multivariate projection methods. First, the data was explored with principal component analysis, PCA, which compresses the data into scores, ***T***, and loadings, ***P***. The decomposition into principal components can be written


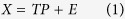


**Figure 3:**
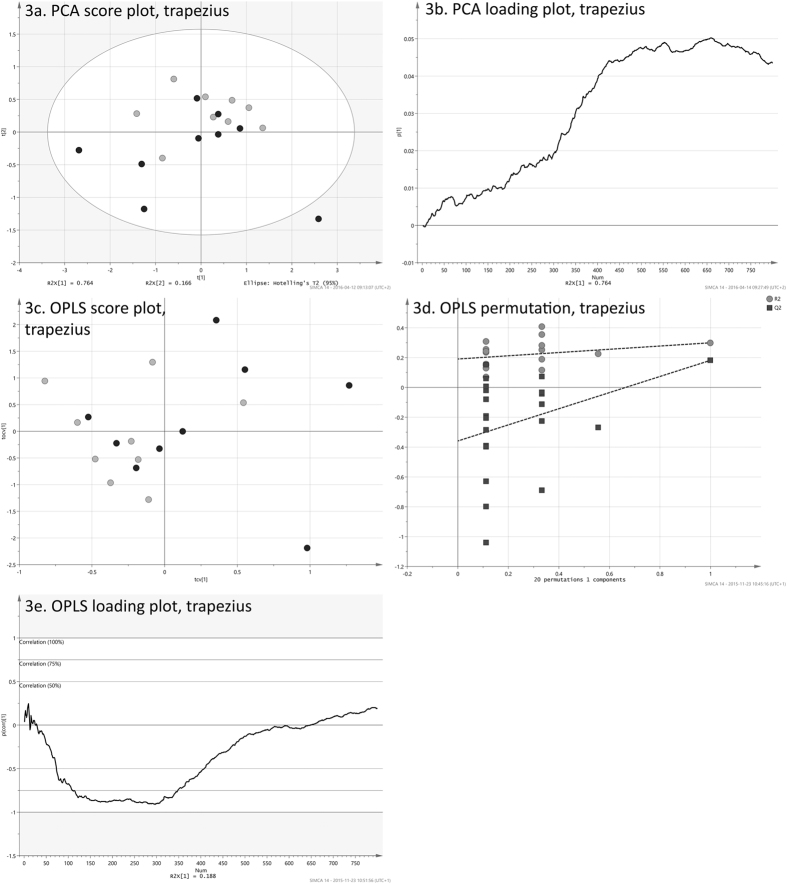
Multivariate analysis of the trapezius muscle. (**a**) The plot illustrates the variation of the 800 strain values, which are compressed into two principal components, t1 (x-axis) and t2 (y-axis). Patient observations, in black, are shifted more to the lower left, compared to healthy controls, in gray. (**b**) Loading plot for the PCA. Nearly all values are positive. These are correlating positively to the observations in the previous score plot, which have positive values t1. As these mostly are healthy controls, it can be presumed that individuals with WAD are showing more shortening in the trapezius muscle during the exercise. (**c**) Cross-validated score plot of the OPLS model created between the 800 deformation values of each individual to the patient status y-variable (patient = 1, control = 0). Controls have predicted score values that are more to the left. Thirteen out of 18 individuals are correctly classified. The OPLS y-correlated score is shown on the x-axis and the orthogonal component on the y-axis. (**d**) Permutation validation of the OPLS model. The plot shows R^2^ (model fit) and Q^2^ (predicted model fit) of 20 models using permuted y-vectors. The x-axis shows the correlation coefficient between the original y and the permuted y-vectors. The y-axis shows the R^2^ and Q^2^ values. The striped lines are regression lines for the R^2^ and Q^2^ values. The dot farthest to the right is the original model (correlation is 1.0). The models with the weakest correlations (~0.12) are found farthest to the left. The models with only a slight correlation have lower Q^2^ values and also a regression line for Q^2^ with steeper inclination. This means the original model is not likely to be found by chance and appears to be valid. (**e**) The correlated deformation loading of the OPLS model, created between the 800 strain values of each patient, and their patient statuses (WAD/control). The plot will show the difference in deformation between the WAD group and the healthy controls over the full dynamic neck extension. The values are scaled as correlation coefficients between the deformation data and the y-correlated score.

**Figure 4:**
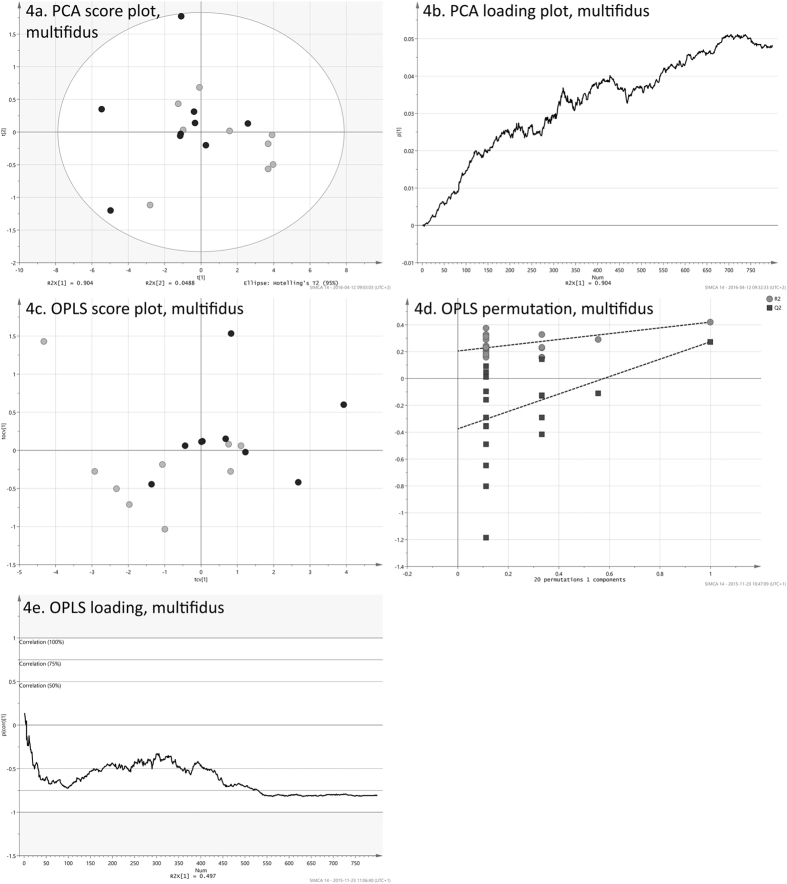
Multivariate analysis of the multifidus muscle. (**a**) The plot illustrates the variation of the 800 strain values, which are compressed into two principal components, t1 (x-axis) and t2 (y-axis). Patient observations, in black, are shifted more to the lower left, compared to healthy controls, in gray. (**b**) Loading plot for the PCA. Nearly all values are positive. These are correlating positively to the observations in the previous score plot, which have positive values t1. As these mostly are healthy controls, it can be presumed that individuals with WAD are showing more shortening in the trapezius muscle during the exercise. (**c**) Cross-validated score plot of the OPLS model created between the 800 strain values of each individual to the patient status y-variable (patient = 1, control = 0). Controls have predicted score values that are more to the left. Thirteen out of 18 individuals are correctly classified. The OPLS y-correlated score is shown on the x-axis and the orthogonal component on the y-axis. (**d**) Permutation validation of the OPLS model. The plot shows R^2^ (model fit) and Q^2^ (predicted model fit) of 20 models using permuted y-vectors. The x-axis shows the correlation coefficient between the original y and the permuted y-vectors. The y-axis shows the R^2^ and Q^2^ values. The striped lines are regression lines for the R^2^ and Q^2^ values. The dot farthest to the right is the original model (correlation is 1.0). The models with the weakest correlations (~0.12) are found farthest to the left. The models with only a slight correlation have lower Q^2^ values and also a regression line for Q^2^ with steeper inclination. This means the original model is not likely to be found by chance and appears to be valid. (**e**) The correlated strain loading of the OPLS model, created between the 800 strain values of each patient, and their patient statuses (WAD/control). The plot will show the difference in strain between the WAD group and the healthy controls over the full dynamic neck extension. The values are scaled as correlation coefficients between the deformation data and the y-correlated score.

The score vectors, ***T***, can be visualized in scatter plots, which gives an overview of the entire data. Each point in a scatter plot corresponds to an observation, which in this case is an individual. The score plot can reveal differences between the observations and it can be possible to find formations of similar observations. The loadings, ***P***, can be used for interpreting the scores. Each score vector will have a corresponding loading vector, which has a value for each of the analyzed variables, i.e. 800. The loading line plot will show the underlying change in deformation, frame by frame, for the variation seen in the actual component. The visual appearance of loading plots can be exemplified by Figs 3b and 4b. Since PCA is an unsupervised method, and will not take into account any variation seen in an external response, its loadings do not necessarily need to reflect the patient status (WAD/control). However, if a score is showing a difference between these, the corresponding loading may be used for interpretation. Scores of positive values will correlate positively to loadings of positive values and they will correlate negatively to negative loading values. On the other hand, scores of negative values will correlate positively to negative loading values and vice versa.

In addition to PCA, the five data matrices were subjected to multivariate regression, where models were created between the deformation sequences of the 18 observations and the patient status (WAD or control). The regression technique used was orthogonal partial least squares, OPLS, which removes variation in ***X*** that is orthogonal to ***y***. The OPLS decomposition of ***X***, the deformation sequence data, and ***y***, i.e. the patient status, can be written


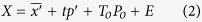



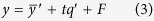


The OPLS algorithm decomposes ***X*** into a single y-correlated component, *t*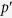 and into one or more *y*-orthogonal components ***T***_***o***_***P***_***o***_. The final PLS model can then be fitted with the correlated component, ***tq***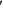. Mean vectors of ***X*** and *y* are given by 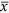 and 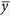. The vector ***t*** and matrix ***T***_***o***_ are scores. Scores correspond to observations, so the number of values in each vector is equivalent to the number of individuals, which in this case is 18. Vectors ***p***, ***q*** and matrix ***P***_***o***_ are loadings. For each existing variable, i.e. analyzed movie frames, a loading value is obtained, which means a loading vector will in this case have a length of 800. While scores, ***T***, in general will show similarities or differences between observations, loadings, ***P***, will show the underlying signal that is causing the differences. Contrary to PCA, OPLS creates a supervised modelling between deformations ***X*** and patient status ***y***. Its loading in the correlated component, ***p***, is therefore directly interpretable as the underlying difference between WAD individuals and healthy controls.

The strength of the OPLS model can be assessed by its R^2^Y and Q^2^Y values, where the former denotes explained model variation and the later variation that can be explained by cross-validation. R^2^Y and Q^2^Y values of 1.0 indicates a perfect model. As the study was of a limited size, the OPLS models were internally validated by leave-one-out cross-validation. The cross-validated score values for each the five models, 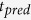, were used as input for statistical testing using the Mann-Whitney-Wilcoxon non-parametric test. A p-value of ≤0.05 was considered to indicate statistical significance when comparing the patients to the control group. The models were further validated by permutation testing. It involves the calculation of a larger number of computed models, which use randomly permuted ***y*** vectors with varying correlation to the original ***y***. Each model is cross-validated and hence R^2^Y and Q^2^Y values can be obtained. These are plotted on the y-axis against the correlation on the x-axis, where the original *y* is farthest to the right. It is expected that the models for the permuted ***y*** vectors generally have lower R^2^Y and Q^2^Y values compared to the original model. Regression lines for the R^2^Y and Q^2^Y distributions can be used for visualizing the trend from less correlated to high correlated ***y***-vectors.

The **y**-correlated loadings of the five OPLS models, one model for each dorsal muscle, were plotted against the 800 movie frames, see Figs 3e and 4e for examples. As the ***y***-correlated loadings explicitly shows the difference between the two groups, these line plots of what could be defined as the *strain loading* made it possible to review the differences between patients and controls over the full neck extension.

As no previous study has compared deformation of dorsal neck muscles during a standardized low-loaded dynamic neck extension in WAD compared with healthy the sample size was arbitrary estimated.

## Results

### Multivariate data analyses of strain sequences

The raw data for all five muscle layers was visualized as line plots (Fig. 2a–e). For each participant, the entire strain sequence, as given by the 800 frames, was represented by one of the plotted lines. The sequence will frame by frame give the strain values over the entire neck extension, and as such, its visualization enabled assessment of all deformation values at once.

It was however difficult to discern any differences between WAD individuals (in black) and healthy controls (in light gray) by means of visual inspection of the strain sequences. The matrices for the five dorsal muscles, comprising full strain sequences, were explored with PCA in order to assess overall differences between patient and control groups for the dynamic neck extension. PCA was here used as an unsupervised tool to illustrate differences between the observations, taking into account all variation found in the strain data and its 800 variables. The first four principal components of all five muscles were visually inspected to find possible differences between the two groups. Of all combinations of scores, only plots for the first two components of the trapezius (Fig. 3a) and multifidus muscle (Fig. 4a) exhibit a distinguishable difference between patient and controls groups. These pairs of components explain 93% and 91% respectively. Although the two groups are not completely separated, both plots have WAD patient observations (in black) positioned more to the left. There are no obvious outliers in the two data sets, as all observations resided inside the 95% confidence regions, given by the Hotelling’s T2 ellipses. The PCA loading line plots of trapezius and multifidus (Figs 3b and 4b), are showing the composition of the first component of the model for the respective muscle. Both exhibited a behavior of increasing strain during the course of the exercise. With 76.4% and 90.4%, these two components are explaining the majority of the variation found in the original data sets, i.e. strain sequences. The loading line plots will show the underlying change in strain, frame by frame, for the variation seen in the actual component. As the two loadings almost only have positive values, these are correlating positively to the observations in the score plots (Figs 3a and 4a), which have positive values in the first component. It can be presumed that individuals with WAD are showing more shortening in the trapezius and multifidus muscles during the exercise as healthy controls correlated positively to the higher strain given by the loading plots. Individuals with WAD are mostly residing the negative side of the two plots, and are hence correlating negatively to the patterns described by the two loading plots.

**Figure 5:**
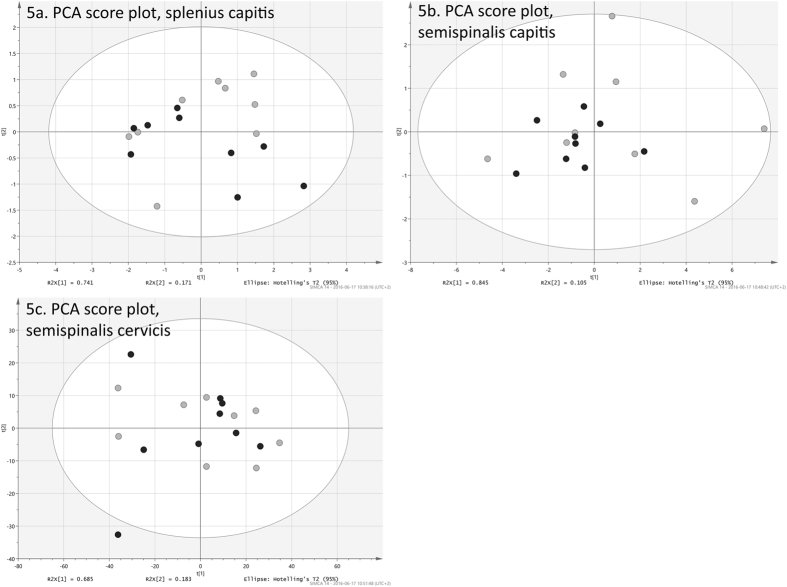
Score scatter plots from the PCA analysis of splenius capitis, semispinalis capitis, semispinalis cervicis. (**a**) PCA scatter plot of splenius capitis. WAD observations are shown in black and controls in light gray. (**b**) PCA scatter plot of semispinalis capitis. WAD observations are shown in black and controls in light gray. (**c**) PCA scatter plot of semispinalis cervicis. WAD observations are shown in black and controls in light gray. *The authors own the intellectual property of all material. The back of the woman (impossible to identify) in* Fig. 1a *belongs to the second author*.

The score plots for the other muscles (splenius capitis, semispinalis capitis, semispinalis cervicis) are shown in Fig. 5. These three exhibited a pattern where no obvious difference between WAD patients and controls could be observed.

To further investigate the found differences, the matrices for the trapezius and multifidus muscles were subjected to OPLS regression. The model found for the trapezius muscle had one orthogonal and one correlated component. The predictive power of the model, given by its Q^2^Y value, was 0.18. The cross-validation yielded 13 out of 18 correctly classified individuals. The cross-validated score plot (Fig. 3c) shows a clear difference between WAD patients (in black) and controls (in light gray), where the controls mostly populate the left side of the plot. Using the correlated scores and their class belongings in a Mann-Whitney-Wilcoxon test, this difference can be translated into a p-value of 0.03. Also, a permutation test (Fig. 3d) shows that model is valid. The corresponding OPLS model for the multifidus muscle, comprising one orthogonal and one correlated component, had a higher Q^2^Y value of 0.27. Again, 13 out of 18 individuals were correctly classified during the cross-validation. It may be pointed out that these 13 correctly classified observations are not automatically the same as the 13 correctly classified for the trapezius muscle. The cross-validated score plot (Fig. 4c) for the multifidus muscle shows an even greater difference between individuals with WAD and controls, where the most of the former are positioned to the right. This difference, using Mann-Whitney-Wilcoxon, can be translated in a highly significant p-value of <0.01. The permutation test for the OPLS model (Fig. 4d) shows that the multifidus model is valid.

The correlated loadings for the two models, Figs 3e and 4e, are reflecting the difference between the WAD and control groups for the dynamic neck extension. As the input to the OPLS models were the full strain sequences, the loadings will frame per frame represent the overall difference between the two groups.

During the neck extension phase (first half part of the dynamic head extension task before returning to neutral), the WAD group showed more shortening in the trapezius muscles compared to the control group (Fig. 3e). The overall difference between the two groups, as given by Fig. 3e, can be translated into a p-value of 0.03. The multifidus muscles in the WAD group had more shortening during both the neck extension phase and the return to neutral part (from neutral position to 20° neck extension and back to neutral position again) compared to the control group (Fig. 4e). These differences, as given by Fig. 4e, can be translated into a p-value of <0.01. There were no significant differences between the WAD and control groups in the other three dorsal neck muscles (p > 0.05).

## Discussion

### Comparison between patients and healthy controls and clinical application

While the visualizations of the sequences were convenient to assess the strain of a single participant over the entire exercise (Fig. 2a–e), it was difficult to tell the difference between WAD individuals (in black) and healthy controls (in light grey). Instead, the multivariate principal component analysis (PCA) and orthogonal partial least squares (OPLS) techniques enabled a comprehensive analysis of the dynamic neck extension exercise for the patient and control groups. With these methods, it was possible to examine the entire exercise, as given by the 800-frame strain sequences. This is in stark contrast to previous studies^5^,^6^,^16^, where these kinds of data were typically reduced to root mean square (RMS) values, areas under or over curves, or to a sum of these. The loadings of the OPLS models, each consisting of 800 values and hence reflecting the entire exercise, were used to interpret the significant differences between the two groups. OPLS loadings, and also PCA loadings for that matter, will correspond exactly to the original variables, i.e. the 800 frames with strain values. These methods create a summary of all variation found in the data. In the case of OPLS, the *strain loading* (Figs 3e and 4e), can be seen as the underlying signal, which separates WAD individuals from healthy controls.

The efficiency of the OPLS models were primarily given by the Q^2^Y values, which denote the prediction efficiency. In many cases, a Q^2^Y value close to unity is not attainable as this scenario would indicate a near flawless prediction. For each of the models, the **y**-variable in the present paper was a “dummy vector” of ones (patients) and zeroes (controls). As the intra-class variation was considerable, as shown by the PCA score plots (Figs 3a and 4a), it follows that the assigned y-values were not perfect. Hence, even with a full separation of the two classes in the score plot, it would not be possible to achieve a Q^2^Y value close to unity.

The WAD group had a significantly different pattern of strain compared with the control group in the most superficial (trapezius) and deepest (multifidus) muscles. The altered pattern in the WAD group consisted of more muscle shortening in the neck extension phase of the exercise. In addition, more multifidus shortening was needed in the return to neutral phase in the WAD group compared with controls. A speculative explanation for the altered pattern of the trapezius and multifidus muscles in the WAD group may be that the subjects need more muscle contraction and effort compared with the controls to resist and control the low load during motion, with the consequence of relative overload (continuous activation without rest) and faster exhaustion of the muscles. Another explanation may be different intervertebral kinematics between healthy and controls with increased motion in the cervical spine in the WAD group^27^. The cervical spine depend on muscle function for support^28^, especially the multifidus, attached with fibers to the cervical facet joint^29^ is important to control intersegmental motion and postural control^28^,^30^. A disturbance in multifidus function may overload the joint and capsule leading to pain. Independent of the speculative explanations of the findings the results indicate an altered and probably ineffective muscle function in the WAD group. This may also be an explanation for the problems remaining after the injury with pain and exhaustion when performing daily and leisure activities^11^,^37^. The study by Peolsson *et al*.^16^ investigated the same seated neck extension in patients with cervical disc disease, compared with healthy controls, using RMS values and reported a reduced strain during the exercise sequence in the semispinalis capitis muscle in patients with cervical disc disease. This was interpreted to be of clinical importance^16^ as the semispinalis capitis is the main neck extensor muscle^31^,^32^ and is capable of exerting large extensor moments to the neck. It may also contribute to functional limitations in neck pain patients, for example when working in prolonged flexed neck positions such as when reading. In the present study, this result was not confirmed. However, the present study provided more advanced analysis of the entire exercise sequence (and the use of a trigger synchronizing the neck device to the ultrasound machine) that enabled splitting the total deformation into shortening and elongation, and this may explain the different results.

During whiplash injury in a car crash, it is suggested that muscle fascicles of the dorsal neck muscles lengthen during the rebound phase^33^, and may lead to muscle strain injuries. It is also known that chronic trauma-induced neck pain patients have more fatty infiltration in their dorsal neck muscles^2^,^3^, have an altered isometric dorsal neck muscle activation pattern^4^,^34^, and a lower isometric endurance of the dorsal neck muscles^7^. Altered activation of the dorsal and ventral neck muscles in neck pain patients has also been reported by several research groups^8^,^13^,^14^,^15^,^35^, with less interplay between the different dorsal and ventral muscle layers during repeated arm exercises in WAD subjects compared with healthy controls^8^,^35^. Less thickness of the multifidus muscle has also been reported during isometric shoulder contraction in neck pain patients compared with healthy controls^36^, supporting earlier findings^5^,^6^,^13^,^14^ of altered ventral neck muscle capability in neck pain patients during arm movements. To the best of our knowledge, the different layers of dorsal neck muscles have not yet been investigated during a dynamic standardised low-loaded neck extension in WAD patients. The present findings, which are in line with other reports of disturbed motor function^5^,^6^,^14^,^15^, may add to the clinical knowledge of alterations in muscle patterning for WAD patients and provide additional support for the importance of retraining muscle function when having persistent musculoskeletal problems after a whiplash event. Recently Ludvigsson *et al*.^11^ and Peterson *et al*.^8^, in a randomised controlled multi-centre trial, showed neck-specific exercises with neuro-muscular control and endurance exercises were favourable over more general exercises. Neck-specific exercises were shown to be more beneficial than no intervention in individuals with chronic WAD^37^. Future studies need to investigate if such an exercise program has a positive effect on the deformation of different muscle layers, leading to a strain pattern more similar to healthy individuals.

### Study limitations

This study has some limitations. One area of concern is the small sample size. The two models for the trapezius and multifidus muscles both had prediction outcomes, which could be translated into significant differences between the patient and control groups. Also, the models were validated by permutation testing, which showed that it is unlikely to find the relationships by chance.

Furthermore, the WAD group was consecutively recruited when fulfilling the criteria. However, all women in the WAD group were carefully interviewed and examined before investigation by experienced physiotherapists and age and gender matched controls were used, with no significant differences in weight and height. Sitting in the neck device lowered the risk of different performances between groups versus the manual resistance used by Peolsson *et al*.^38^. This provided the opportunity to show statistically significant differences in the behaviour of the dorsal neck muscles in patients compared with healthy controls in both the most superficial and deepest dorsal neck muscles investigated. Another difference from the study by Peolsson *et al*.^16^ was a trigger was used at the head cushion to synchronise the neck device with the ultrasound machine, although an improvement from the study by Peolsson *et al*.^16^, the motion angle was still not captured.

The advantage of using ultrasound movies with speckle tracking analysis is that different muscle layers can be examined non-invasively in real time during the entire exercise sequence. The length of each muscle, the location of the origin and insertion, and the location of the force during the dynamic neck extension are all factors that can influence the results. Unfortunately, these factors cannot be measured or controlled for with the ultrasound measurements used. In addition, the rotational movements of muscles cannot be measured during movement as the ultrasound captures two-dimensional movements. The different muscle layers may also have been imaged in different planes from each other and the architecture and position of the muscle may have been altered due to pathological or length changes.

Although a standardised load was used, there could be differences between participants in how many percentages of maximal voluntary capacity they need to use when performing the dynamic neck extension. The results of the present study provide information on how the muscles behave during a low loaded task but not in relation to every single participant’s own maximal capacity. Although Lopata *et al*.^25^ indicated a relationship between muscle deformation obtained with speckle tracking analysis and force, muscle function is complex and it is not straightforward to equalise deformation with muscle strength or muscle activity. Muscle strain obtained by ultrasound movies describes the mechanical shortening and elongation of the muscle during an exercise and not the electric action potential, as in EMG; however, the ultrasound investigation makes it possible to non-invasively capture multilayers of muscles. Compared with magnetic resonance imaging ultrasound capture muscles during real time and the entire exercise performance. To increase the understanding of the entire exercise strain as well as during the two different phases, we analysed the entire exercise sequence. Despite the limitations of the present study, it adds new valuable information on patients with WAD for clinical practice and this may impact future rehabilitation. Additionally, the neck muscle behaviour registered with ultrasonography has been analysed during the entire exercise with a new multivariate method, making it possible to evaluate the complete dorsal muscle strain sequence, comprising hundreds of continuous values.

## Conclusions

The present study provides preliminary evidence of altered mechanical strain of the trapezius and multifidus muscles in individuals with WAD, compared with healthy controls, when performing a standardised low-loaded neck extension. Although there were significant findings in 2 of 5 muscles, the results need to be interpreted with some caution due to the small sample size. The findings may be of importance for the future diagnosis and treatment of individuals with chronic WAD.

## Additional Information

**How to cite this article**: Peolsson, A. *et al*. Multivariate analysis of ultrasound-recorded dorsal strain sequences: Investigation of dynamic neck extensions in women with chronic whiplash associated disorders. *Sci. Rep*. **6**, 30415; doi: 10.1038/srep30415 (2016).
